# Natural products in atherosclerosis therapy by targeting PPARs: a review focusing on lipid metabolism and inflammation

**DOI:** 10.3389/fcvm.2024.1372055

**Published:** 2024-04-18

**Authors:** Yan Zhang, Xue-Ying Zhang, Shan-Rui Shi, Chao-Nan Ma, Yun-Peng Lin, Wen-Gang Song, Shou-Dong Guo

**Affiliations:** ^1^ Department of Endocrinology and Metabolism, Guiqian International General Hospital, Guiyang, China; ^2^ Institute of Lipid Metabolism and Atherosclerosis, School of Pharmacy, Shandong Second Medical University, Weifang, China; ^3^ Department of General Surgery, Qixia Traditional Chinese Medicine Hospital in Shandong Province, Yantai, China; ^4^ Shandong Provincial Key Laboratory for Rheumatic Disease and Translational Medicine, The First Affiliated Hospital of Shandong First Medical University & Shandong Provincial Qianfoshan Hospital, Jinan, China

**Keywords:** PPARα, PPARβ, PPARγ, lipid metabolism, inflammation, cardiovascular disease

## Abstract

Inflammation and dyslipidemia are critical inducing factors of atherosclerosis. Peroxisome proliferator-activated receptors (PPARs) are ligand-activated transcription factors and control the expression of multiple genes that are involved in lipid metabolism and inflammatory responses. However, synthesized PPAR agonists exhibit contrary therapeutic effects and various side effects in atherosclerosis therapy. Natural products are structural diversity and have a good safety. Recent studies find that natural herbs and compounds exhibit attractive therapeutic effects on atherosclerosis by alleviating hyperlipidemia and inflammation through modulation of PPARs. Importantly, the preparation of natural products generally causes significantly lower environmental pollution compared to that of synthesized chemical compounds. Therefore, it is interesting to discover novel PPAR modulator and develop alternative strategies for atherosclerosis therapy based on natural herbs and compounds. This article reviews recent findings, mainly from the year of 2020 to present, about the roles of natural herbs and compounds in regulation of PPARs and their therapeutic effects on atherosclerosis. This article provides alternative strategies and theoretical basis for atherosclerosis therapy using natural herbs and compounds by targeting PPARs, and offers valuable information for researchers that are interested in developing novel PPAR modulators.

## 1 Introduction

Cardiovascular disease (CVD) has become the number one cause of human death due to changes in lifestyle, especially high-fat and high-caloric diet, and aging of population. It is estimated that approximately 170,000 people die from CVD each year (1). Of note, atherosclerosis is an important cause of CVD events (2). In the year of 2020, nearly 2 billion people suffered from carotid atherosclerosis in the world including 270 million people in China (3, 4). Atherosclerosis is a chronic inflammatory and degenerative process that primarily occurs in large- and medium-sized arteries. This disease is characterized by accumulation of fatty and fibrous materials and calcium minerals in the intima layer of arteries (5, 6).

Inflammation drives all phases of atherosclerosis including initiation, metaphase, advanced phase, and rupture or regression (6). Thus, inflammatory factors, such as C-reactive protein (CRP), interleukin (IL)-6, and tumor necrosis factor-α (TNF-α), are consistently elevated in atherosclerosis. Furthermore, receptors and other molecules involved in inflammation, such as toll-like receptor (TLR), particularly, TLR2 and TLR4, are augmented in human atherosclerotic plaques **(**7, 8). Dyslipidemia, characterized by high levels of total cholesterol (TC) and triglyceride (TG) and low levels of high-density lipoprotein (HDL) cholesterol (HDL-c), is equally or even more dangerous for the onset and development of atherosclerosis. It is acknowledged that low-density lipoprotein (LDL) cholesterol (LDL-c) or LDL particles and hypertriglyceridemia or TG-rich lipoproteins are leading inducing factors of atherosclerosis (9, 10).

Peroxisome proliferator-activated receptors (PPARs) are recognized as promoters of peroxisome proliferation more than 40 years ago (11). Due to their various functions, research on PPARs has grown exponentially in recent years. Notably, the distribution and function of PPARs exhibit organ- and cell-specificity. PPARα is chiefly expressed in heart, liver, skeletal muscle, and cardiovascular system; PPARβ/δ is widely distributed in the body; and PPARγ is highly expressed in white adipose tissue (12–14). The roles of PPARs in physiological and pathological conditions have been reviewed recently by distinct groups (14–16). Mechanistically, PPARs heterodimerize with retinoid X receptor (RXR) and bind to specific DNA regions of target genes (AGGTCAXAGGTCA, with X being a random nucleotide) that are termed as peroxisome proliferator hormone response elements. Ligand activation triggers conformational changes of PPAR-RXR and finally activate the transcription of target genes. Notably, PPARs regulate multiple genes associated with cellular lipid metabolism and inflammation in cardiovascular system (14). Downregulation of PPARα is found to decrease hepatic *de novo* lipogenesis, while PPARα agonists restore lipid homeostasis in the liver (17). Mechanistically, PPARα induces the expression of genes involved in fatty acid uptake, conversion, and catabolism through β-oxidation pathway, leading to reductions in fatty acid and TG synthesis and hepatic very low-density lipoprotein production. Similar to PPARα, PPARβ activates carnitine palmitoyl transferase (CPT), which facilitates fatty acid transport across mitochondrial membrane and the subsequent β-oxidation **(**18). Furthermore, PPARβ activation enhances energy expenditure through upregulation of heat-producing enzymes including uncoupling protein 1 and 3 in brown adipose tissue, thereby protecting against obesity and fatty liver. On the contrary, PPARγ agonists, such as rosiglitazone, cannot decrease TG and fatty acid levels. Mechanistically, PPARγ increases glucose utilization, thereby decreasing glucose–fatty acid cycle and the subsequent upregulation of the genes involved in fatty acid synthesis and uptake (19).

Moreover, activated PPARs can interact with other transcription factors that are involved in inflammation, such as activator protein 1 (AP-1) and nuclear factor kappa B (NF-κB), resulting in transcriptional repression (14, 20). For instance, PPARα activation suppresses inflammatory responses in different cells by inhibiting TLR4/NF-κB and AP-1 signaling pathways (14, 17–22). PPARβ is demonstrated to decrease inflammation via activation of AMP-activated protein kinase (AMPK) and inactivation of mitogen-activated protein kinase (MAPK) signaling pathways. However, deletion or repression of PPARβ expression in myeloid cells also decreases atherosclerosis and inflammatory molecules by modulating the PPARβ/B cell lymphoma 6 axis (14, 23). Moreover, PPARγ activation has been demonstrated to inhibit release of inflammatory factors via activating AMPK and suppressing multiple signaling pathways including TLR4, MAPK, and WNT/β-catenin (14). Therefore, PPARs are considered as important targets for CVD therapy and other diseases.

In a previous article, we reviewed PPARs' regulation and their roles in atherosclerosis as well as synthesized PPAR agonists and antagonists **(**14). Although synthetic PPAR modulators exhibit attractive potential in atherosclerosis therapy, these compounds induce various side effects and show contrary therapeutic effects in different participants and animal models. Notably, phytochemical compounds show therapeutic effects in different diseases by modulation of PPARs (24, 25), and they are considered as preventive agents for metabolic syndrome including nonalcoholic fatty liver disease (NAFLD) by targeting PPARs (26, 27). Given multiple diseases, particularly NAFLD, diabetes, obesity, and fibrosis, are closely associated with the onset and development of atherosclerosis (28–32), compounds with the activities of ameliorating the above diseases are useful for atherosclerosis therapy. Importantly, the majority of natural products exhibit good therapeutic efficacy and safety compared to synthetic medications **(**15, 33). These properties suggest that natural products are potential candidate molecules for atherosclerosis therapy. This article reviews the roles of natural herbs and compounds in treatment of atherosclerosis through activation of PPARs by focusing on lipid metabolism and inflammation. Recent literatures, mainly from 2020 to present, published in PubMed, Web of Science, and Google Scholar were screened out using traditional Chinese medicine (TCM), flavonoid, acid, alkaloid, terpenoid, phenolic compound, and carbohydrate in combination with PPAR as key words.

## 2 TCM in regulation of PPARs

### 2.1 TCM prescription and lipid metabolism

**Figure 1:**
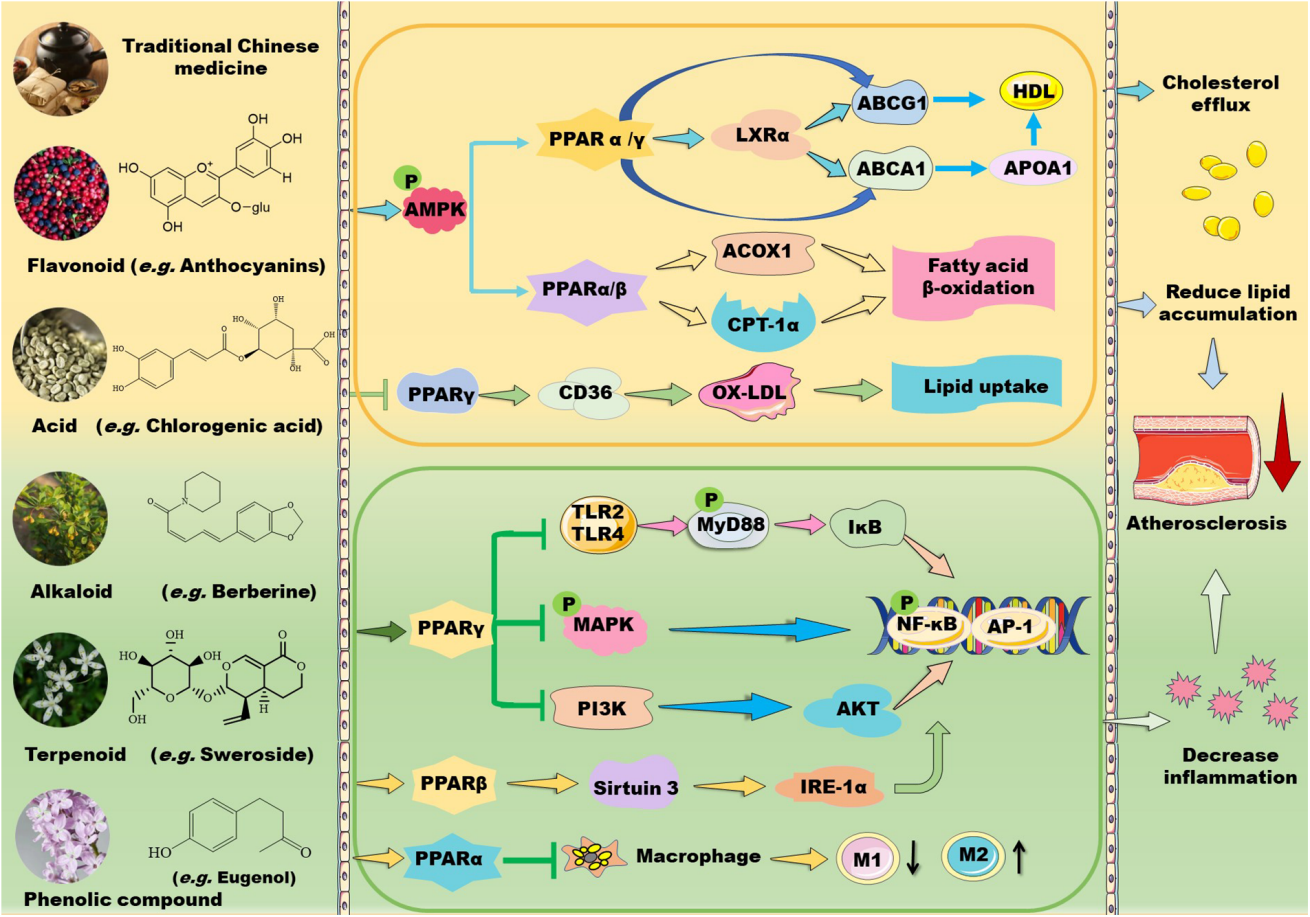
Mechanisms of action of TCM prescriptions and natural bioactive molecules in atherosclerosis therapy by targeting peroxisome proliferator-activated receptors (PPARs). TCM prescriptions and natural bioactive molecules including flavonoids, natural acids, alkaloids, terpenoids, and phenolic compounds mainly decreases lipid accumulation by activating AMP-activated protein kinase (AMPK) and the subsequent signaling pathways including PPARα/carnitine palmitoyl transferase (CPT)-1 and acyl-CoA oxidase 1 (ACOX1)-mediated fatty acid β-oxidation in liver and PPARγ/liver X receptor (LXR) α/ATP-binding cassette transporter (ABC) A1/ABCG1-mediated cholesterol efflux from macrophages to apolipoprotein (Apo) A1 and high-density lipoprotein (HDL) particles, thereby decreasing foam cell formation. Moreover, some TCM prescriptions and natural molecules may decrease cluster of differentiation (CD) 36-mediated lipid absorption via suppressing PPARγ, thereby reducing lipid accumulation in macrophages. Notably, TCM prescriptions and natural molecules primarily ameliorate inflammation by suppressing mitogen-activated protein kinase (MAPK)/nuclear factor kappa B (NK-κB) and phosphoinositide-3 kinase (PI3K)/protein kinase B (AKT/PKB)/NK-κB signaling pathways through activation of PPARγ. Furthermore, these natural compounds can inhibit Toll-like receptor (TLR)4/myeloid differentiation factor 88 (MyD88)/NF-κB signaling pathway and promote macrophage shift to an anti-inflammatory M2 type through activation of PPARα. Notably, natural compounds may stimulate PPARγ coactivator (PGC)-1β–estrogen related receptor α to activate PPARβ/PPARγ signaling pathways and enhance protein kinase A (PKA)/AMPK signaling pathway to upregulate PPARα in the liver. Except for NF-κB, nuclear transcription factor activator protein 1 (AP-1) is involved in the modulatory effects of PPARs on anti-inflammation. These beneficial effects of TCMs are supposed to retard the development of atherosclerosis. IKK: inhibitor of nuclear factor κB kinase subunit; IL: interleukin; TNF-α: tumor necrosis factor α.

TCMs have been used for treatment of metabolic disorders and CVDs for hundreds of years*.* Recent studies have demonstrated that TCMs ameliorate hyperlipidemia and atherosclerosis through modulation of PPARs (Figure 1). Huo-Xue-Qu-Yu formula (HXQY, 活血祛瘀方) ameliorates lipid profiles including apolipoprotein (Apo) B and ApoA1 in rats with NAFLD via upregulating the expression of PPARα and CPT-1 in the liver, thereby improving symptoms of NAFLD (34). Similarly, heart-protecting musk pill (麝香护心丸) is found to attenuate atherosclerosis partially via activating PPARα/CPT-1α signaling pathway in ApoE-deficient mice (35). TCM believes that “phlegm stasis interjunction (痰瘀互结)” is an important inducing factor in the occurrence and development of atherosclerosis. Dan-Lou prescription (丹蒌方) has been demonstrated to reduce phlegm, repair diseased blood vessels, and eliminate hyperlipidemia, thus ameliorating atherosclerosis. Notably, this prescription enhances cholesterol efflux by activating PPARα/ATP-binding cassette transporter (ABC) A1 signaling pathway (36).

In addition to activating PPARα signaling, many TCM prescriptions stimulate PPARγ–liver X receptor (LXR) α–ABCA1/ABCG1 signaling pathways, thereby ameliorating lipid profiles and atherosclerosis through upregulation of reverse cholesterol transport (RCT). Qi-Huang-Zhu-Yu Formula (QHZY, 岐黄茱萸方) enhances cholesterol efflux by activating PPARγ–LXRα–ABCA1/ABCG1 signaling pathways (37). Notably, Qing-Xue-Xiao-Zhi formula (QXXZ, 清血消脂方), Si-Ni decoction (四逆汤), Qi-Shen-Yi-Qi Pill (芪参益气丸), and Yin-Xing-Tong-Mai decoction (银杏通脉汤) have been demonstrated to attenuate hyperlipidemia and atherosclerosis by facilitating RCT through upregulation of PPARγ–LXRα–ABCA1/ABCG1 signaling pathways (38–41). Moreover, Dang-Gui-Shao-Yao-San (当归芍药散), a well-known Chinese medicine formula, improves lipid metabolism and inhibits neuroinflammation by activating LXR–PPARγ signaling pathway (42). However, Hua-Tan-Jiang-Zhuo decoction (化痰降浊汤) alleviates TC and TG levels mainly by inhibiting the gene expression of PPARγ, cholesterol 7-α-hydroxylase A1, and sterol response element-binding protein (SREBP)-1c in hyperlipidemic rats (43).

### 2.2 TCM prescription and inflammation

TCMs also suppress inflammation by modulating PPAR signaling pathways (Figure 1). Shen-Tong-Zhu-Yu decoction (参通逐瘀汤) reduces secretion of pro-inflammatory cytokines, such as IL-1β, IL-6, and TNF-α in rheumatoid arthritis fibroblast-like synoviocytes. Mechanistically, this decoction inhibits phosphorylation of p38 MAPK and activates PPARγ, thereby modulating the p38-MAPK/PPARγ signaling pathway (44). Shen-Hong-Tong-Luo Formula (参红通络方) has been used in clinic for more than 30 years in China. This formula inhibits reactive oxygen species (ROS) accumulation and reverses lipopolysaccharide (LPS)- and oxidized LDL-induced inflammation and lipid accumulation in macrophages by activating PPARγ/LXRα/ABCA1 pathway (45). *Schisandra sphenanthera* improves liver steatosis and inflammation via activating PPARα/γ signaling in C57BL/6J mice with NAFLD (46). Compound Dan-Shen Dripping Pill (CDDP, 复方丹参滴丸) or QHZY alleviates inflammation via modulating PPARγ/NF-κB p65 signaling pathway (37, 47). The major anti-atherosclerotic components of Compound Dan-Shen formula are ginsenoside Rg1, notoginsenoside R1, and protocatechuic aldehyde; these molecules inhibit endothelial cell damage via suppressing focal adhesion kinase (FAK)-phosphatidylinositol 3-kinase (PI3K)/protein kinase B (PKB/AKT) signaling pathway (48). In ApoE-deficient mice, QXXZ inhibits inflammation by suppressing TLR4-myeloid differentiation factor 88 (MyD88)-NF-κB signaling pathway (38). Notably, Bu-Shen-Kang-Shuai formula and Tan-Yu-Tong-Zhi formula ameliorate atherosclerosis potentially via promoting macrophage polarization towards an M2 phenotype through activation of PPARγ and downregulation of NK-κB (49, 50). However, *Gynostemma pentaphyllum*, a TCM that is generally used to treat hypercholesterolemia and inflammation, has been demonstrated to reduce obesity and obesity-related inflammation by down-regulating PPARγ signaling pathway (51).

## 3 Natural compounds in regulation of PPARs

### 3.1 Flavonoids in regulation of PPARs

**Figure 2:**
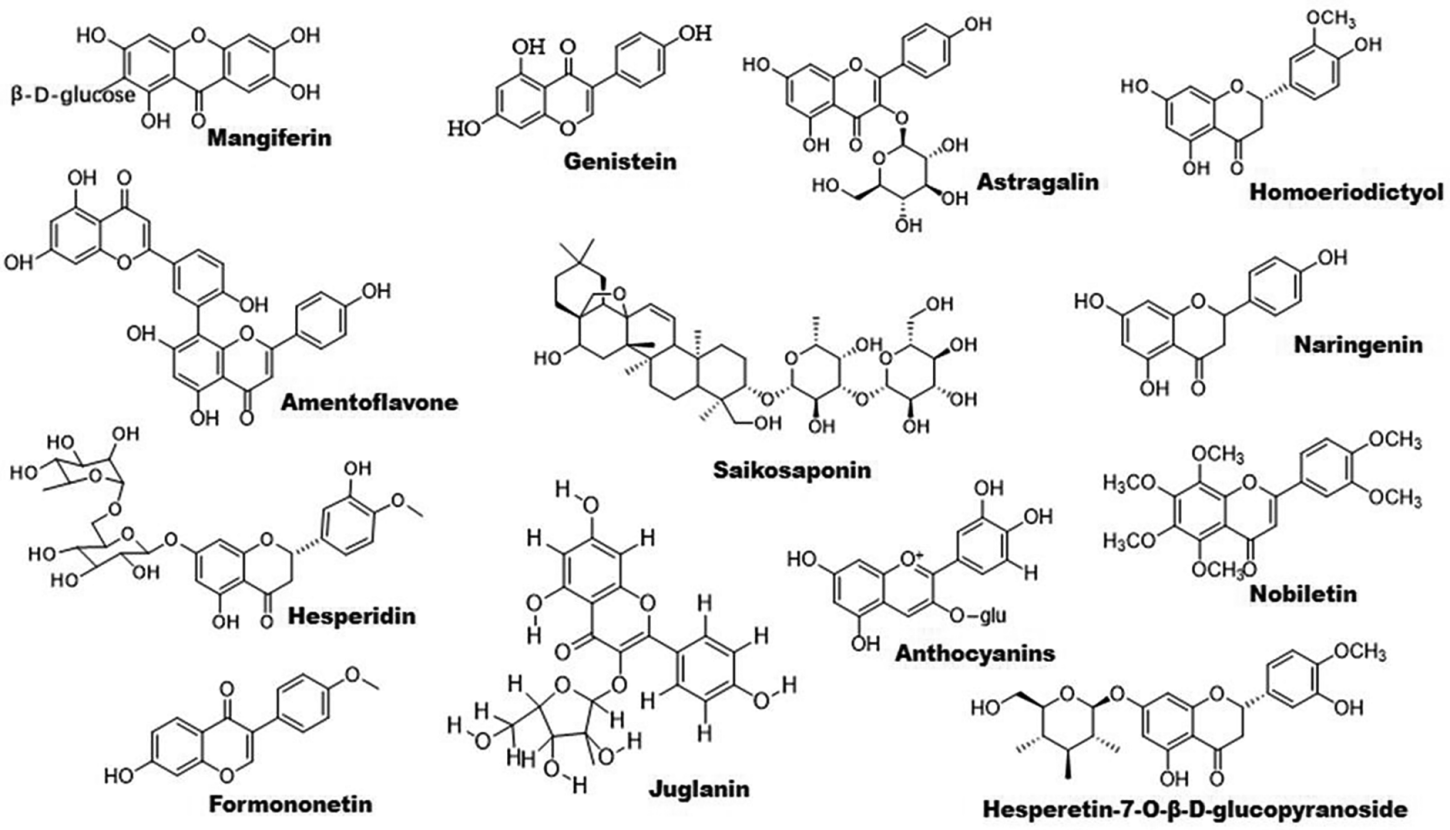
Chemical structures of some bioactive flavonoids with potential anti-atherosclerotic effects.

Plants-derived flavonoids have been demonstrated to improve lipid metabolism and inflammation by modulating PPAR signaling pathways (Figures 1, 2). These natural flavonoids provide a new therapeutic direction for treatment of atherosclerosis. The anti-inflammatory and anti-allergic potential as well as the basic structure of some dietary flavonoids have been reviewed recently by Rakha et al. (52). Moreover, the CVD-protecting effects of myricetin have been summarized in the literature (53).

#### 3.1.1 Flavonoids and lipid metabolism

Citrus flavonoids play an important role in treatment of dyslipidemia and atherosclerosis. The roles of Citrus fruits-derived compounds in modulation of metabolic diseases have been reviewed recently by Aslan et al. (54). Nobiletin is an active component of citrus peel. This molecule increases the expression of PPARγ but not PPARα. Furthermore, it activates AMPK, thus promoting the expression of ABC transporters including ABCA1 and ABCG1. Notably, the LXRα–PPARγ loop amplifies its action **(**55). *Rosa rugosa* Thunb- and *Rosa davurica* Pall. fruits-derived flavonoids upregulate the expression of PPARα and its downstream genes that are involved in lipid metabolism (56, 57). Genestein improves lipid metabolism by upregulating PPARα and activating estrogen receptor β-AKT-mammalian target of rapamycin (mTOR) signaling pathway (58). Hesperidin decreases TG levels by enhancing PPARα and suppressing PPARγ and other lipogenic genes including SREBP-1, fatty acid synthesis (FAS), and stearoyl-CoA desaturase; it reduces TC by suppressing cholesterol absorption through downregulation of fatty acid binding protein (FABP) and retinol binding protein (59).

Flavonoids are found to improve RCT by activating PPARγ/PPARα–ABC transporter pathway. For example, sea buckthorn flavonoids improve hyperlipidemia by up-regulating PPARα/CPT-1α and PPARγ/ABCA1 signaling pathways (53). Total flavonoid extract obtained from *Psoralea corylifolia* L. seeds alleviates oxidized-LDL-induced foam cell formation via enhancing PPARγ–ABCA1/ABCG1 signaling pathways *in vitro* and in LDLR-deficient mice (60). In line with these findings, other compounds including mangiferin, quercetin, astragalin, and biochanin A have been demonstrated to ameliorate atherosclerosis via enhancing macrophage cholesterol efflux and RCT through activation of the PPARγ–LXRα–ABCA1/ABCG1 signaling pathways (61–66). Similarly, *S. baicalensis*-derived flavonoids and baicalein regulates glucose and lipid homeostasis through upregulation of AMPK/PPARγ/LXRα signaling pathway (67, 68). Homoeriodictyol and hesperidin-7-O-β-D-glucopyranoside are found to significantly increase the level of PPARγ protein, providing new candidates for treatment of atherosclerosis (69). Interestingly, amentoflavone prevents oxidized-LDL-induced lipid accumulation by suppressing PPARγ/cluster of differentiation (CD) 36-mediated lipid uptake (70). It is worth noting that several flavonoids exhibit powerful lipid-lowering effects in clinical studies as reviewed recently Gouveia et al. (71).

#### 3.1.2 Flavonoids and inflammation

Accumulating evidence have demonstrated that some flavonoids reduce inflammation by regulating PPAR signaling pathway (Figure 1). Formononetin, an Astragalus-derived isoflavone, inhibits inflammation by reducing the release of proinflammatory cytokines (72). Furthermore, it reduces oxidized-LDL-induced endothelial injury by stimulating PPARγ signaling pathway, contributing to its anti-atherosclerotic effects (9). Biochanin A activates PPARγ/LXRα/ABC transporter and PPARγ/heme oxygenase 1 signaling pathways to suppress hyperlipidemia-induced inflammation in ApoE-deficient mice (65). Similarly, astragalin stimulates PPARγ–LXRα–ABCA1/ABCG1 signaling pathways, which in turn suppress TLR4/NF-κB signaling pathway, thereby inhibiting inflammation in foam cells (64). Propolis-derived flavonoids reduce inflammatory cytokines and endoplasmic reticulum (ER) stress by activating PPARγ in a myocardial infarction model (73). Saikosaponin A and anthocyanins decrease the release of pro-inflammatory cytokines by activating PPARγ, thereby suppressing the NF-κB signaling pathway (74, 75). However, genistein reverses Ang II-induced downregulation of PPARγ to inhibit the expression of CRP and matrix metalloproteinase 9 in vascular smooth muscle cells (VSMCs), thereby reducing inflammatory responses in atherosclerosis **(**76).

### 3.2 Natural acids in regulation of PPARs

#### 3.2.1 Natural acids and lipid metabolism

**Figure 3:**
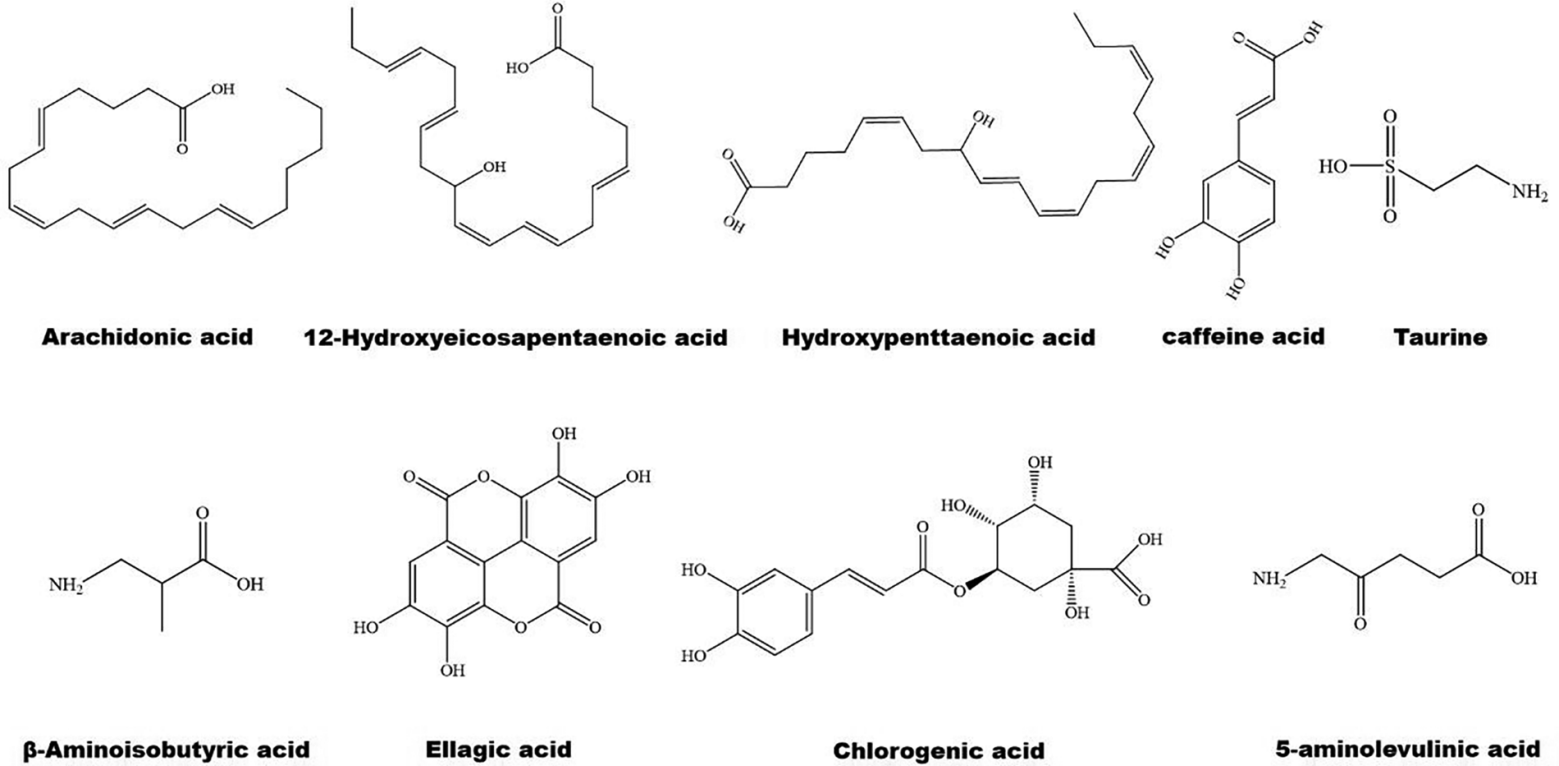
The structure of some bioactive acids with potential anti-atherosclerotic effects.

The structure of some bioactive natural acids and their mechanisms of action are shown in Figures 1, 3. The widely distributed chlorogenic acid and caffeine acid are demonstrated to benefit health and cardiovascular system **(**77). The anti-obesity properties of chlorogenic acid have been recently reviewed by Kumar et al. (78). Notably, chlorogenic acid and caffeine acid may act synergistically on reducing lipid deposition in macrophages via inhibiting PPARγ signaling pathway (77). Furthermore, 5-aminolevulinic acid-mediated sonodynamic therapy improves cholesterol efflux via activating PPARγ–LXRα–ABCA1/ABCG1 signaling pathways, enhancing efferocytosis and cholesterol efflux, and eventually ameliorating atherosclerosis **(**79).

Oleic acid prevents intracellular lipid accumulation in human macrophages through modulation of PPARs and down-regulation of ApoB48 receptor, suggesting the role of monounsaturated fatty acid in regulation of postprandial TG-rich lipoprotein/ApoB48 receptor axis (80). Dodecahexaenoic acid (DHA) ameliorates postprandial hyperlipidemia potentially by upregulating PPARα and the genes involved in fatty acid β-oxidation and down-regulating TG and ApoB secretion (81). Furthermore, *ω*-3 polyunsaturated fatty acids (PUFAs) attenuate hepatic steatosis through upregulation of PPARα/CPT-1α signaling pathway **(**82). Supplement of DHA-rich fish oil increases PPARγ activity in peripheral blood mononuclear cells of the participants (83). However, administration of DHA rapidly increases the production of cyclic adenosine monophosphate inside cilia, and finally activates PPARγ to initiate adipogenesis in preadipocytes **(**84).

Hydroxypentaenoic acid reduces LDL-c levels and increases HDL-c levels in atherosclerotic animal models, leading to reductions in aortic atherosclerotic plaques (85–87). Mechanistically, this molecule acts as a PPAR ligand and elevates LXRs–ABCA1/ABCG1 signaling pathways (85, 88). Similarly, 12-Hydroxyeicosapentaenoic acid reduces foam cell formation and atherosclerosis via activation of PPARγ–ABCA1/ABCG1 signaling pathways **(**89, 90). 8-hydroxyeicosapentaenoic acid is a pan PPAR activator and has beneficial effects against dyslipidemia and atherosclerosis **(**86). However, medium-chain structured lipids ameliorate high-fat diet-induced atherosclerosis potentially by reducing the expression of PPARγ **(**91). It seems that carbon number of fatty acids plays a role in regulation of PPARγ.

#### 3.2.2 Natural acids and inflammation

Notably, arachidonic acid acts as an activator of PPARα (92) and has a therapeutic effect on atherosclerosis (93). Taurine is one of the most abundant arachidonic acid in animals. It counteracts chronic inflammation in adipose tissues potentially via promoting macrophage polarization toward an anti-inflammatory M2 phenotype (94). Similarly, 12-Hydroxyeicosapentaenoic acid promotes macrophage shift towards an anti-inflammatory M2 phenotype (90, 95), thereby inhibiting atherosclerosis (96). β-aminoisobutyric acid protects against vascular inflammation via upregulating PPARγ coactivator (PGC)-1β–estrogen related receptor α–PPARβ/PPARγ signaling pathways (97). As reviewed recently, amino acid derivatives may alleviate inflammation and improve energy expenditure and obesity by targeting PPARs (98). Furthermore, PUFAs are involved in resolution of inflammation (99, 100). Dietary *ω*-3 and *ω*-6 PUFAs upregulate gene expression of PPARs, thereby suppressing inflammation and lipid accumulation (101). For instance, both PPARγ and PPARα can be activated by *ω*-3 PUFAs (102). Unfortunately, *ω*-3 PUFAs show limited effects on CVD events in clinical trials (9).

### 3.3 Alkaloids in regulation of PPARs

#### 3.3.1 Alkaloids and lipid metabolism

**Figure 4:**
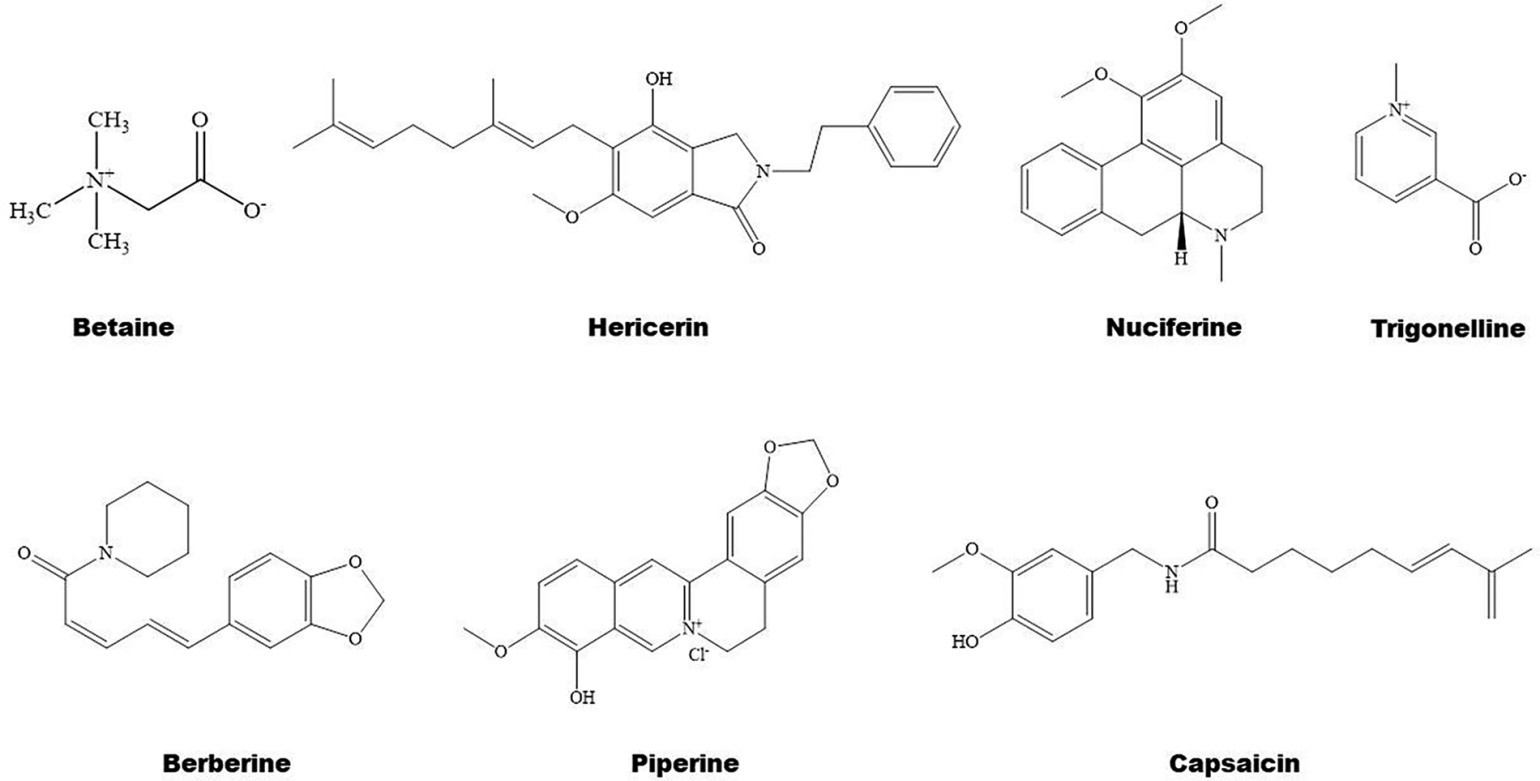
Structure of some bioactive alkaloids with potential anti-atherosclerotic effects.

Some natural alkaloids and their derivatives are reported to be PPAR modulators (Figures 1, 4). For instance, (S)-tryptophan-betaxanthin and berberrubine are demonstrated to be leading compounds of pan PPAR activators based on a screening of 30,000 TCM candidates (103). Berberine, an isoquinoline alkaloid, has been used for treatment of CVDs as reviewed recently by Song et al. **(**104). In diabetic atherosclerosis, berberine stimulates Krüppel-like factor 16/PPARα signaling pathway, thereby improving lipid metabolism (105). In adipose tissue, berberine activates AMPK/Sirtuin 1 axis, an energy metabolic sensing pathway, increasing PPARγ deacetylation, thereby promoting adipose tissue remodeling and thermogenesis through upregulation of uncoupling protein 1 (106). In liver, berberine treatment increases lipid oxidation by upregulating PPARα and its downstream genes including CPT-1α and acyl-CoA oxidase 1 (ACOX1) (107). *In vitro*, berberine and its major metabolite berberrubine attenuate lipid accumulation in HepG2 cells via upregulating PPARα signaling pathway (108). Similarly, the protective effect of theobromine against NAFLD is partially attributed to its upregulation of PPARα and CPT-1α (109). Furthermore, nuciferine improves hepatic steatosis by activating PPARα/PGC-1α pathway in diabetic mice (110). Betaine attenuates hyperlipidemia by activating PPARα and PPARγ and their downstream gene LXRα (111). Hericerin, an indolinone meroterpenoid alkaloid, has been defined as a strong PPARγ agonist with potential hypoglycemic and hypolipidemic effects **(**112). Coffee-derived trigonelline, an alkaloid derivative of niacin (vitamin B3), alleviates hyperlipidemia by increasing PPARα and decreasing PPARγ expression (113). Additionally, capsaicin may suppress obesity by suppressing PPARγ signaling pathway **(**42, 114).

#### 3.3.2 Alkaloids and inflammation

Berberine treatment suppresses systemic inflammation by reducing the production of inflammatory factors including TNF-α and LPS through activation of PPARα and its potential target thyroid hormone responsive (107). The anti-inflammatory mechanisms of betaine are associated with inhibition of TLR4/NF-κB signaling pathway and regulation of PPARs (115). Furthermore, betaine alleviates high-fat diet-induced inflammation by modulating silent information regulator 1/SREBP1/PPARα signaling pathway, thereby suppressing the expression of NF-κB (116). Capsaicin inhibits oxidized-LDL-induced ROS generation and VSMC phenotypic switching by activating PPARα (117), and ameliorates diabetic retinopathy by suppressing PPARγ–poldip2–NADPH oxidase 4 signaling pathway **(**118). Interestingly, the anti-inflammatory effect of capsaicin is LXRα–PPARγ dependent **(**119). Moreover, trigonelline exhibits antioxidant and anti-inflammatory effects partially by activation of PPARα (113, 120), piperine inhibits cardiac fibrosis via activating PPARγ and the following inhibition of AKT/glycogen synthase 3β signaling pathway (121), and nuciferine suppresses myocardial injury by upregulating PPARγ in mice **(**122).

### 3.4 Terpenoids in regulation of PPARs

#### 3.4.1 Terpenoids and lipid metabolism

**Figure 5:**
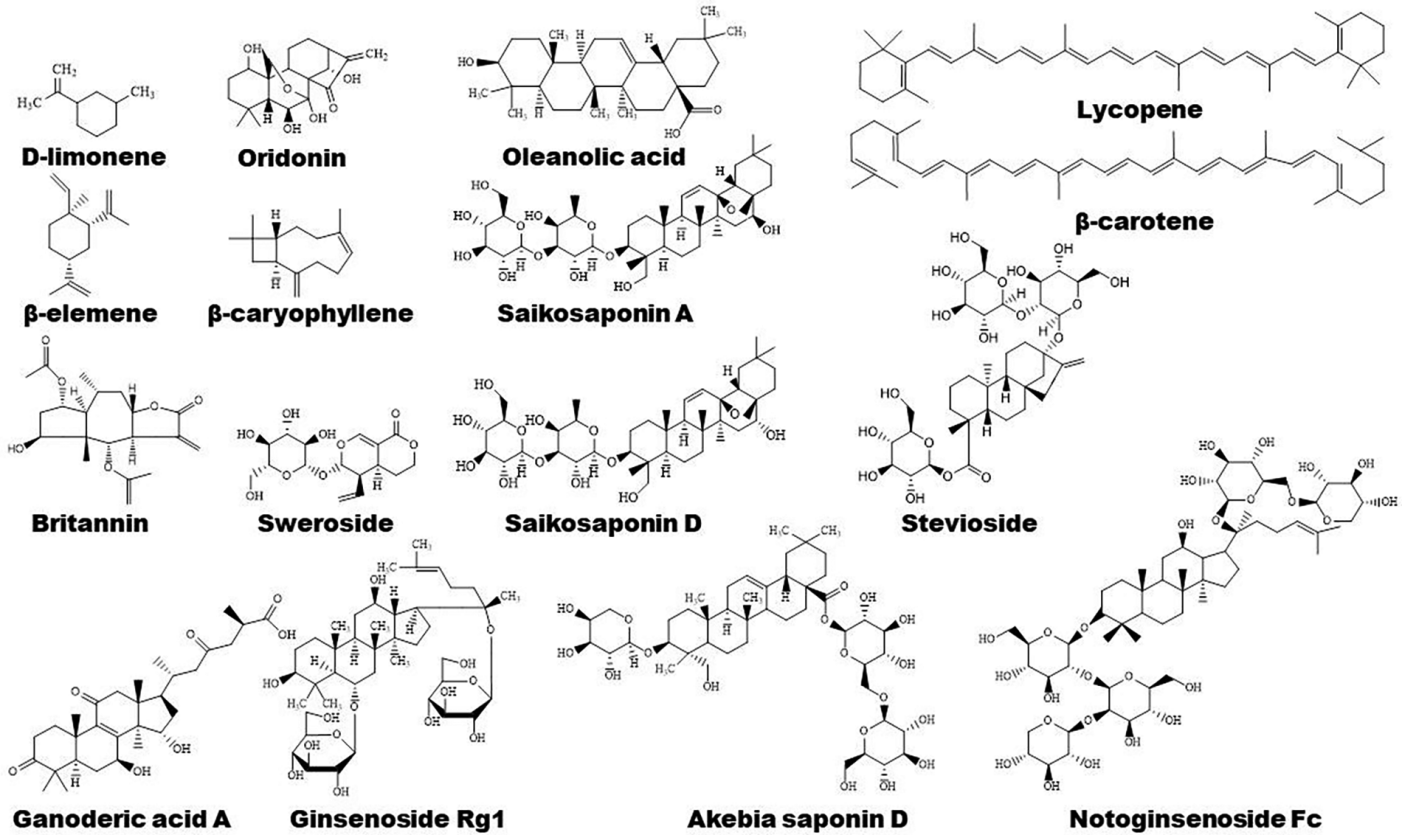
Structure of some bioactive terpenoids with potential anti-atherosclerotic effects.

Terpenoids are found to ameliorate hyperlipidemia by targeting PPARs (Figures 1, 5). Eugenol, a phenolic monoterpenoid, increases the expression of PPARα, partially contributing to its hypolipidemic and antioxidant properties in diabetic rats **(**123). Sweroside protects against obesity mainly by enhancing PPARα (124). Ginsenoside Rg1, baicalin, and *Resina Commiphora*-derived terpenoids also improve lipid metabolism and atherosclerosis through upregulation of PPARα and its target genes including CPT-1 and ACOX1 (125–127). Saikosaponin D and diosgenin serve as PPARα agonists, promoting PPARα-mediated fatty acid oxidation and inhibiting CD36-mediated fatty acid uptake and SREBP-1c-mediated *de novo* lipogenesis (128, 129). Oleanolic acid, a pentacyclic triterpenoid, and (E)-β-caryophyllene, a bicyclic sesquiterpene hydrocarbon, act as dual activator of PPARα and PPARγ, decreasing hyperglycemia and lipid accumulation (130, 131). Furthermore, Saikosaponin A and ginsenoside 20(R/S)-Rg3 act as natural PPARγ activators, ameliorating hyperlipidemia and atherosclerosis (72, 132). However, ginsenoside Rg1 inhibits lipid uptake via downregulation of PPARγ (125), Ganoderic acid A suppresses oxidized-LDL-induced lipid accumulation in THP-1-derived macrophages by inhibiting Notch1-PPARγ-CD36 signaling pathway (111), and D-limonene, decreases lipid anabolism by decreasing the expression of PPARγ and SREBP-1c, and activating the AMPK signaling pathway in high-calorie diet-induced obese rats (133).

Sea cucumber saponins reduce lipogenesis and promote fatty acid β-oxidation via inhibiting SREBP-1c and enhancing the expression of PPARα and ACOX1, respectively, thereby improving lipid deposition in rodents **(**134–137). In combination with eicosapentaenoic acid-enriched phospholipids, sea cucumber saponins further reduce hepatic TG partially by enhancing the expression of PPARα as reviewed by Lin et al. (137). Interestingly, sea cucumber saponin treatment induces changes of lipid metabolism-related genes including PPARα in rhythm, suggesting saponin may modulate lipid metabolism by regulating the clock genes, such as CLOCK and BMAL1 (137, 138). The major bioactive component of saponin, echinoside A, also regulates the expression of some key genes that are involved in lipid metabolism in a diurnal manner (139). The marine-derived PPAR activators have been reviewed recently by D'Aniello et al. (140).

#### 3.4.2 Terpenoids and inflammation

PPARγ plays a vital role in anti-inflammatory mechanisms of action of terpenoids (Figure 1). Saponin notoginsenoside Fc ameliorates inflammatory response in high glucose-induced endothelial cell injury partly by activation of PPARγ (141). Stevioside attenuates inflammation by upregulating PPARγ, thereby activating PI3K/AKT signaling pathway in a middle cerebral artery occlusion/reperfusion rat model (142). Ginsenoside Rg3 represses FAK-mediated expression of vascular cell adhesion molecule (VCAM)-1 and intercellular cell adhesion molecule (ICAM)-1 through activation of PPARγ (143). Saikosaponin A or britannin decreases inflammation potentially by activating PPARγ, thereby downregulating NF-κB signaling pathway (74, 144). Furthermore, lycopene, β-carotene, and oridonin may act as PPARγ modulators (145, 146). Interestingly, geraniol, an acyclic mono-terpenoid alcohol, decreases LPS/interferon γ-induced NLRP3 inflammasome activation and macrophage M1-type polarization through inhibiting PPARγ methylation (147). However, Akebia saponin D is reported to ameliorate high-fat diet-induced gut barrier injury via repressing PPARγ-FABP4 signaling pathway (110). Additionally, β-elemene augments the mRNA expression of PPARβ and CPT-1α and sirtuin 3, thereby blocking lipid-induced inflammatory pathways (148).

### 3.5 Phenolic compounds in regulation of PPARs

#### 3.5.1 Phenolic compounds and lipid metabolism

**Figure 6:**
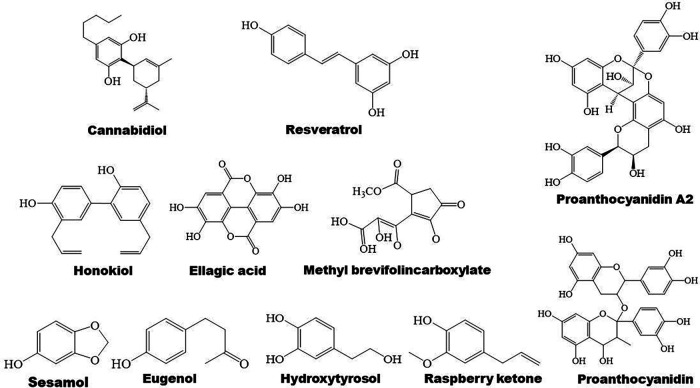
Structure of some bioactive phenolic compounds with potential anti-atherosclerotic effects.

Phenolic compounds are widely distributed bioactive compounds, they are found to exert lipid-modulatory and anti-inflammatory functions by regulating PPARs (Figures 1, 6). Resveratrol ameliorates hepatocyte steatosis via activating protein kinase A/AMPK/PPARα signaling pathway (149). It abolishes intestinal fatty acid and monoglyceride accumulation via activation of PPARα/PPARγ and their downstream ABCA1 and ABCG1 transporters in atherosclerotic mice (150). Furthermore, it is found to promote fatty acid β-oxidation by enhancing MAPK/PPAR signaling pathway (151). Polydatin, the glucoside of resveratrol, activates PPARβ signaling pathway to improve lipid metabolism (152). Raspberry ketone increases phosphorylation of AMPK to improve fatty acid oxidation through upregulation of PPARα and CPT-1 **(**153).

Ellagic acid has anti-atherogenic and cardioprotective properties, suggesting its role in atherosclerosis therapy **(**154). Mechanistically, ellagic acid regulates the genes that are mainly correlated with PPAR signaling pathway, thereby ameliorating lipid metabolism (155). Hydroxytyrosol, a polyphenol, decreases the expression of FAS, SREBP-1c, and PPARγ, ameliorating TC and TG levels and hepatic steatosis in ethanol-induced HepG2 cells (156). In addition, methyl brevifolincarboxylate, a polyphenolic compound, improves hepatic lipid accumulation through upregulation of AMPKα/PPARα signaling pathway and the target genes of PPARα including CPT-1 and ACOX1 in free fatty acid-treated hepatocytes (157). Sesamol, a phenolic compound derived from sesame oil, activates PPAR signaling pathway, leading to enhanced fatty acid oxidation, cholesterol efflux, and catabolism, thus accelerating lipid consumption and reducing intracellular lipid accumulation in HepG2 cells (158).

#### 3.5.2 Phenolic compounds and inflammation

Proanthocyanidin A2 and ellagic acid exhibit anti-inflammatory properties potentially by upregulating PPARγ signaling pathway (159, 160). Cannabidiol, a nonpsychoactive cannabinoid, inhibits inflammation through downregulation of TLR4/NLRP3/Caspase-1 signaling pathway in a PPARγ-dependent manner in Caco-2 cells **(**161). Furthermore, cannabidiol might exert anti-inflammatory effects by either directly or indirectly modulating PPARγ/NF-κB/nuclear factor erythroid 2-related factor 2 signaling in urothelial cells (162). Honokiol dramatically reduces production of proinflammatory cytokines in mice with ulcerative colitis that is induced by dextran sulfate sodium (DSS) partially via upregulating PPARγ and suppressing TLR4/NF-κB signaling pathway (163). The activation of PPARγ by honokiol is also associated with its effects on preventing against hyperglycemia and CVD (20). Furthermore, resveratrol suppresses hepatic inflammation via activation of PPARγ and downregulation of ER stress-mediated apoptosis (164). Forsythiaside A regulates PPARγ/RXR-α complex, inhibiting TLR4/MAPK/NF-κB and NF-κB/MLCK/MLC2 signal pathways, thus suppressing LPS-induced inflammation and epithelial barrier damages. However, forsythiaside A enhances the expression of PPARγ/RXR-α complex in lung and inhibits this complex in colon, suggesting its cellular-specific effects (165). Additionally, proanthocyanidin alleviates liver ischemia/reperfusion injury by suppressing autophagy and apoptosis through regulation of PPARα/PGC-1α signaling pathway (166). Mechanisms of action of phenolic compounds in regulation of inflammation is concluded in Figure 1.

### 3.6 Carbohydrates in regulation of PPARs

**Figure 7:**
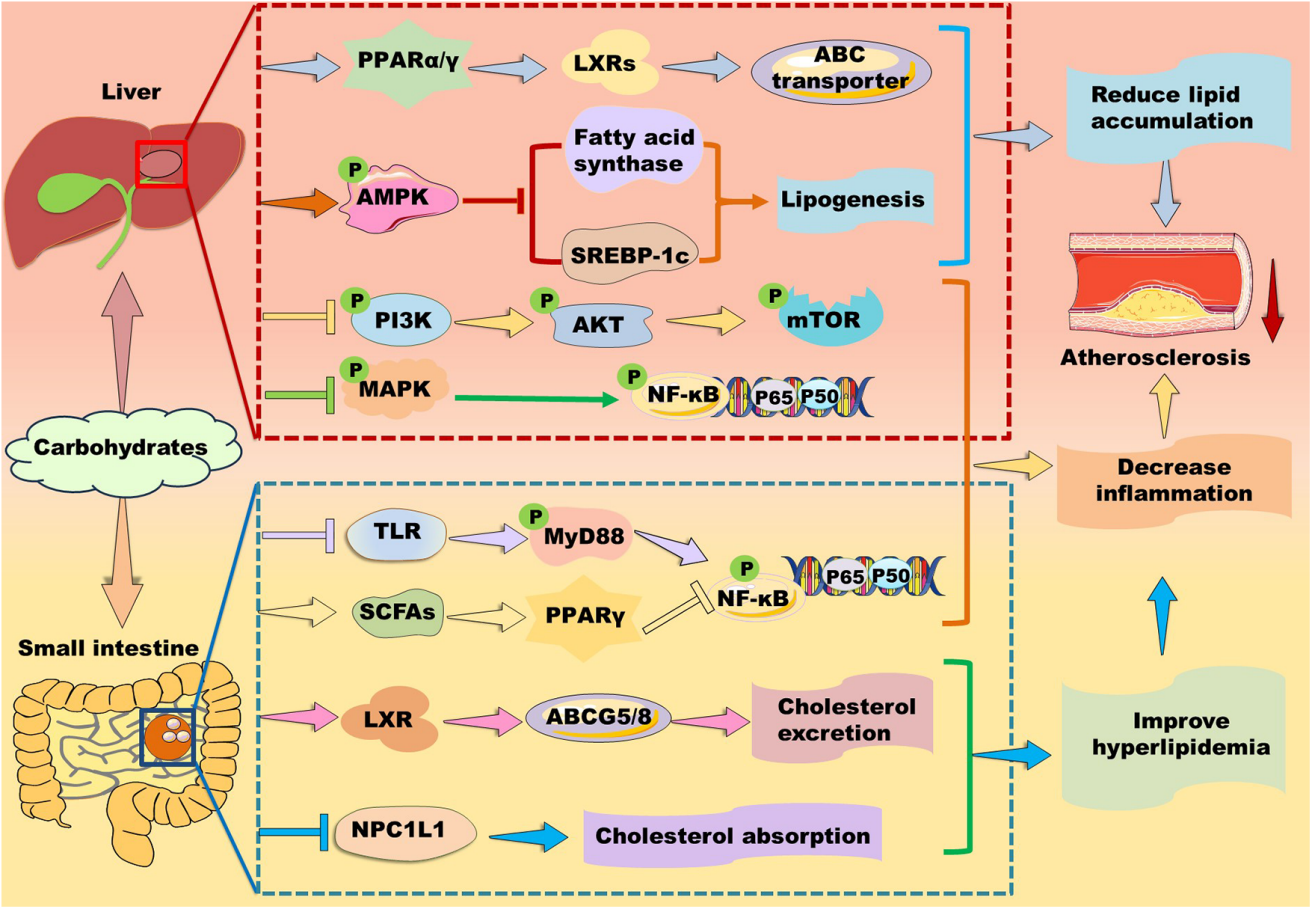
Mechanisms of action of carbohydrates in atherosclerosis therapy. Carbohydrates prevent against lipid accumulation by enhancing PPARα/PPARγ–liver X receptor (LXR)–ABC transporter and PPARα–mediated fatty acid β-oxidation. Alternatively, some polysaccharides alleviate lipid accumulation by suppressing fatty acid synthesis-related genes including PPARγ, fatty acid synthase, and sterol response element-binding protein (SREBP)-1c potentially through up-regulation of AMP-activated protein kinase (AMPK) in the liver. Furthermore, they inhibit phosphoinositide-3 kinase (PI3K)/protein kinase B (AKT/PKB)/mammalian target of rapamycin (mTOR) and mitogen-activated protein kinase (MAPK)/nuclear factor kappa B (NK-κB) signaling pathways to suppress inflammation. In the small intestine, carbohydrates decrease cholesterol absorption and increase cholesterol excretion by decreasing the level of Niemann-Pick C1-like 1 protein and enhancing the LXR/ABCG5/8 signaling pathway, respectively. Moreover, carbohydrates suppress inflammation by inhibiting Toll-like receptor (TLR)/myeloid differentiation factor 88 (MyD88)/NF-κB and modulating PPARγ/NK-κB signaling pathway.

Polysaccharides are a kind of carbohydrate polymers that are generally consisted of more than ten monosaccharides through glycosidic linkages in linear or branched chains. Given polysaccharides generally have low toxicity and various biological activities, such as antioxidant, anti-inflammatory, and anti-atherosclerosis, some polysaccharides have been used in medical and biochemical areas as reviewed by different groups **(**137, 167–169). Notably, carbohydrates are found to exert their function via activating PPAR signaling pathways (Figure 7).

#### 3.6.1 Carbohydrates and lipid metabolism

Different groups have demonstrated that brown seaweed fucoidans attenuate hyperlipidemia and atherosclerosis by modulating PPARs in different animal models (170). For instance, *Kjellmaniella crassifolia*-derived fucoidan ameliorates hyperlipidemia by improving PPARα-mediated fatty acid β-oxidation in Wistar rats (171). Similarly, *Saccharina sculpera*-derived fucoidans improve hyperlipidemia potentially by enhancing the gene expression of PPARα and PPARγ in Wistar rats (172). *Cladosiphon okamuranus*-derived fucoidan improves hyperlipidemia and atherosclerosis partially by elevating the expression of PPARα and inhibiting SREBP-1 (173). Except for PPARα, PPARγ activation also stimulates LXR/ABC transporter signaling pathways, thereby accelerating lipid transport and excretion (14). However, *Ascophyllum nodosum*-derived fucoidan is found to inhibit the expression of PPARγ and elevate the expression of PPARα, thereby attenuating hyperlipidemia and atherosclerosis in ApoE-deficient mice (174).

Besides brown seaweeds, sea cucumber-derived polysaccharides improve lipid metabolism in different models (137). For instance, *Isostihopus badionotus*-derived fucosylated chondroitin sulfate (4,300 Da) exhibits a hypolipidemic effect in mice partially by down-regulating the expression of FAS and PPARγ (175). *Acaudina molpadioides*-derived fucoidan inhibits adipocyte proliferation and differentiation via enhancing Wnt/β-Catenin signaling pathway and suppressing the expression of SREBP-1c and PPARγ (176, 177). Glycosaminoglycans isolated from sea cucumber *Holothuria leucospilota* are found to ameliorate hyperlipidemia in male BALB/c mice by improving the expression of PPARα and ameliorating gut microbiota (137, 178).

Polysaccharides isolated from plants and fungi also exhibit powerful lipid-lowering effects as reviewed recently by distinct groups (179, 180). *Cyclocarya paliurus*-, *Saussurea involucrata*-, *Astragalus membranaceus*-, and *Cordyceps militaris*-derived polysaccharides exert therapeutic effects in hyperlipidemic rats partially via upregulating PPARα/CPT signaling pathway (181–184). A water-soluble polysaccharide from *Morchella esculenta* alleviates obesity and liver injury mainly by restoring Firmicutes/Bacteroidetes ratio and increasing SCFA production. However, it decreases hepatic gene expression including PPARα and PPARγ (185). Similarly, *Liriope spicata* var. prolifera- and *Platycodon grandiflorus*-derived polysaccharides exhibit strong lipid-lowering and hepatoprotective effects potentially by downregulating the expressions of PPARγ *in vivo* (186). Interestingly, *P. grandiflorus*-derived polysaccharides may control PPAR signaling by increasing the production of SCFAs including acetate, propionate, and butyrate in the gut through upregulation of SCFAs-producing gut bacteria (187). Similarly, *Pueraria lobata*- and *Pueraria thomsonii*-derived polysaccharides show therapeutic effects in type 2 diabetes mellitus through regulation of PPAR signaling pathway. Mechanistically, *P. lobata*-derived polysaccharides increase the abundance of *Romboutsia* bacteria to reduce serum concentration of taurocholic acid, thereby regulating the PPAR signaling pathway, such as inhibiting PPARγ. *P. thomsonii*-derived polysaccharides reduce the abundance of *Klebsiella* bacteria to decrease the serum levels of uric acid, thereby regulating PPAR signaling pathway to exert a therapeutic effect on insulin resistance (188). *Lycium barbarum* polysaccharide and Astragalus polysaccharide ameliorate lipid disorders by decreasing the gene expression of PPARγ, CD36, and FAS, and ameliorating gut microbiota **(**189, 190). Moreover, *C. militaris*-derived polysaccharide CM3-SII is demonstrated to inhibit the level of Niemann-Pick C1-like 1 protein, suggesting this polysaccharide may decrease cholesterol absorption (184).

Except for polysaccharide, monosaccharide and oligosaccharide have been demonstrated to modulate lipid metabolism by targeting PPARs. For instance, D-psicose regulates lipid metabolism via stimulating AMPK2α/PPARα signaling in rats (191). D-mannose promotes fatty acid oxidation via enhancing PPARα (192). Our group demonstrates that N-acetylneuraminic acid reduces TC and particularly TG partially by enhancing PPARα in ApoE-deficient mice (193, 194). Aging enhances the expression of SREBP-1c and decreases the expression of PPARα. Interestingly, oral intake of trehalose reverses these changes in aged liver, suggesting trehalose decreases lipogenesis and boosts fatty acid β-oxidation (195). Fructose is considered as a lipogenic nutrient. It suppresses transcriptional activity of PPARα and its target gene CPT-1α, potentially via modulating PGC-1α acetylation and CPT-1α acetylation (196).

#### 3.6.2 Carbohydrates and inflammation

In a comparative study, fucoidans obtained from *Undaria pinnatiﬁda, F. vesiculosus, Macrocystis pyrifera, A. nodosum, and Laminaria japonica* reduce production of pro-inflammatory cytokines in a dose-dependent manner in LPS-induced cells (197). Mechanistically, fucoidans suppress MAPK/NF-κB, Janus kinase/signal transducer and activator of transcription-1/3, and TLR/MyD88/NF-κB signaling pathways (198). Furthermore, sea cucumber *Apostichopus japonicus*-derived fucoidan decreases LPS-induced inflammation by suppressing phosphorylation of p38-MAPK and the downstream NF-κB and AKT/mTOR pathway (176). Moreover, *Sargassum horneri*-derived fucoidan is found to suppress inflammation by inhibiting phosphorylation of p38-MAPK, c-Jun amino-terminal kinases (JNK), and extracellular signal-regulated kinase (ERK) (199). As PPAR activation modulates inflammation-related signaling pathway (14), fucoidans may suppress inflammation partially by regulating the expression of PPARs as mentioned above.

Notably, *L. japonica*-derived fucoidan decreases intestinal inflammation potentially by upregulating PPARα and improving gut microbiota (200), *S. involucrata* polysaccharide alleviates ultraviolet radiation-induced inflammatory responses by activating PPARα **(**183). Furthermore, *L. barbarum* polysaccharide inhibits LPS-induced inflammation by upregulating PPARγ and suppressing phosphorylation of p38-MAPK, JNK, and ERK, suggesting this polysaccharide alleviating inflammatory reactions through modulation of PPARγ/MAPK/NF-κB signaling pathway (201). Similarly, *Moringa oleifera* leaf polysaccharide ameliorates DSS-induced colitis by enhancing PPARγ and decreasing TLR/MyD88/NF-κB signaling pathway (202). Interestingly, polysaccharides may suppress hyperlipidemia-induced inflammation by decreasing PPARγ in hyperlipidemic animals. For instance, *Tibetan burnip* polysaccharide reduces the expression of ICAM-1, VCAM-1, IL-6, IL-1β, and TNF-α partially by downregulating PPARγ in hyperlipidemic rats (203). Moreover, intake of low dose sucrose (7.5 mg/ml) is found to activate PPARγ via restoring microbial dysfunction and upregulating SCFAs levels, thereby suppressing MAPK/NF-κB signaling pathway, while high dose sucrose (30 mg/ml) exacerbates DSS-induced colitis (204). Additionally, Astragalus polysaccharide inhibits protein kinase A/p38 MAPK signaling pathway and the expression of PPARγ and PGC-1α, suppressing inflammation in heart failure rats (190). These data suggest that polysaccharides may control inflammatory response by differently regulating the expression of PPARγ based on the actual situation. Given polysaccharides with big molecular weight and great hydrophilic property are hard to be absorbed, their microbiota-derived metabolites including SCFAs may play a key role in regulation of PPARs and atherosclerotic therapy.

## 4 Concluding remarks and future directions

TCMs, especially TCM prescriptions, and natural compounds including flavonoids, acids, alkaloids, terpenoids, phenolic compounds, and carbohydrates are effective in suppression of dyslipidemia and inflammatory responses with good safety by targeting PPARs, thereby retarding the progression of atherosclerosis. Notably, these natural molecules exhibit equivalent effects compared to chemically synthetic compounds but the former exhibit less harmful side effects (15). Furthermore, TCMs have been used for atherosclerosis therapy for hundreds of years in Asia, especially in China. Importantly, several natural compounds, such as anthocyanins, resveratrol, hesperidin, quercetin, epicatechin, and genistein, have been promoted to clinical trials (71). In this study, we also listed some clinical trials as shown in Table 1. Collectively, natural compounds are useful for atherosclerosis therapy by regulation of PPARs.

**Table 1:** Clinical trials related to anti-atherosclerotic effects of natural medicines.

Compound	Patients and dosage	Effects and potential mechanisms
Anthocyanin	90 patients with prediabetes and 70 newly diagnosed diabetes; twice daily for a total of 320 mg for 12 weeks.	Increases adiponectin in newly diagnosed diabetes potentially by activation of AMPK-PPARα signaling pathway (205).
Anthocyanin	176 subjects aged 35–70 years with dyslipidemia; 40 mg/day, *n* = 45; 80 mg/day, *n* = 42; 320 mg/day, *n* = 43) for 12 weeks.	Ameliorates dyslipidemia, lowers plasma ceramide 16:0 and ceramide 18:0, and increases HDL-c, ApoA-I, and cholesterol efflux capacity potentially by activation of PPAR-ABCA1/G1 signaling pathways (206, 207).
Hesperidin	49 patients with metabolic syndrome; twice daily for a total of 500 mg for 12 weeks.	Decreases fasting glucose level, TG, blood pressure and inflammatory factors including TNF-α potentially by activation of PPARs (208).
Glucosyl hesperidin	Subjects with high-TG type (>150 mg/dl); 500 mg for 24 weeks.	Decreases TG, ApoB, and ApoC particles potentially by improving VLDL metabolic abnormality (209).
Flaxseed power and/or hesperidin	98 patients with metabolic syndrome; flaxseed powder 30 g daily, or hesperidin 1 g daily, or a combination for 12 weeks.	Decreases systolic blood pressure, serum TG and insulin potentially by activating PPARα and regulating ApoB100 secretion (210).
Genistein	45 participants with homeostasis model assessment index >2.5 and body mass index ≥30 and ≤40 kg/m^2^; 50 mg daily for 2 months.	Increases β-oxidation and decreases inflammatory symptoms and insulin resistance potentially by modulating gut microbiota and activating AMPK-PPARs signaling pathway (211).
Omega-3 fatty acid (fish oil)	102 patients with PPARγ gene polymorphisms, LDL-c 70–190 mg/dl and TG ≥ 400 mg/dl; 2 g daily for 3 months.	Decreases LDL-c and TG in carriers of PPARγ polymorphisms, suggesting genetic-driven personalization of cardiovascular interventions (212).
DHA-rich fish oil	Fifty patients with type 2 diabetes mellitus aged 30–70 years; 2,400 mg/d for 8 weeks.	Increases PPAR-γ activity in peripheral blood mononuclear cells (83).
Conjugated linoleic acid	15 healthy human; 90 g daily for 2 or 4 weeks.	Improves n-3 highly unsaturated fatty acids potentially via activating PPARα (213).
Epigallocatechin gallate	Obese subjects older than 18 years; 150 mg daily for 8 weeks.	Decreases plasma TG, blood pressure and kisspeptin levels without impacting PPARγ (214).
Colchicine	4,745 patients recruited within 30 days after a myocardial infarction; 0.5 mg daily.	Decreases risk of ischemic cardiovascular events (215).
Resveratrol	Meta-analysis of 21 randomized clinical trials; 0.1–1.5 g daily for 4–24 weeks.	Decreases TC levels and may increase HDL-c (216).
Ginsenoside Rg1	Random crossover trial (112 type 2 diabetic patients); 41 mg daily for 2 weeks.	Decreases TC, TG, and blood glucose levels potentially by activating PPARγ (217).
Lycopene	126 healthy men; 6 mg (*n* = 41), or 15 mg (*n* = 37) daily for 8 weeks.	Decreases inflammatory factors and increases antioxidant activities (218).
Glucomannan, inulin, psyllium and apple fibre	100 overweight or obese participants; dietary fibres for 8 weeks.	Decreases body mass index, body weight, TC, LDL-c, TG, and C-reactive protein (219).
Barley β-glucan	Fifteen healthy subjects; a soup containing high-molecular-weight barley β-glucan with great viscosity.	Decreases diet-induced thermogenesis and glycaemic response by delaying gastric emptying (220).
*Ascophyllum nodosum*	43 healthy subjects (19 men, 24 women), aged 21–63 years; 0.9 g daily for 6 weeks.	Decreases body weight, body mass index, and potentially TG (221).
*Lycium barbarum* polysaccharide	50 patients with non-alcohol fatty liver disease; twice daily for a total of 0.6 g for 3 months.	Results are not available at present (222).
Polysaccharide peptide of *Ganoderma lucidum*	37 high risk and 34 stable angina patients; three times daily for a total of 0.75 g for 90 days.	Decreases atherosclerosis potentially by decreasing circulating endothelial cells and endothelial progenitor cells, and oxidation as well as malondialdehyde contents (223).
Trehalose	15 patients with history of myocardial infarction and evidence of systemic inflammation; intravenous trehalose (15 g once weekly) for 12 weeks.	No significant reduction in arterial wall inflammation, larger studies are needed (224).

However, the research in this field has several limitations. First, although prescription/formula is a characteristic of TCM, it is necessary to clarify the key active ingredients and their mechanisms of action to enable TCM to enter the international market. In this aspect, artemisinin is a very good example. Secondly, seldom natural compounds have been applied in clinic. It seems that researchers are impelled to explore modified natural compounds to improve their novelty, bioavailability, and commercial value of interested molecules. These chemical modifications are sure to induce further environmental pollution. Therefore, researchers need to balance the beneficial and harmful aspects during drug discovery. Thirdly, as the distribution and action of PPARs show tissue-specificity, it is interesting to investigate the combined effects of interested compounds based on their pharmacokinetic characteristics and tissue distribution. Fourth, both activation and inactivation of PPARβ and particularly PPARγ may achieve similar therapeutic effects, suggesting some complex regulatory mechanisms are involved in PPARs' therapy of atherosclerosis. For instance, PPARγ activation is demonstrated to suppress inflammation via inhibiting NF-κB signaling pathway and decrease lipid accumulation via enhancing RCT through upregulation of LXRs–ABCA1/G1 signaling pathways; while PPARγ inactivation is indicated to decrease lipogenesis and CD36-mediated lipid uptake, thereby suppressing lipid accumulation and hyperlipidemia-induced inflammation. To elucidate the detailed mechanisms of action of an interested compound, it is necessary to investigated the above-mentioned mechanisms in one study in the future. Last but not least, rodents have distinct lipid profiles and lifestyles compared to our human, it is necessary to explore humanized models for drug screening in future to improve the potential translation of interested compounds.
